# Silicone-based highly stretchable multifunctional fiber pumps

**DOI:** 10.1038/s41598-024-55472-0

**Published:** 2024-02-26

**Authors:** Ryo Kanno, Keita Shimizu, Kazuya Murakami, Yuya Shibahara, Naoki Ogawa, Hideko Akai, Jun Shintake

**Affiliations:** 1 Shintake Research Group, Department of Mechanical and Intelligent Systems Engineering, The University of Electro-Communications, 1-5-1 Chofugaoka, Chofu, Tokyo, 182-8585 Japan; 2 Swiss Federal Laboratories for Materials Science and Technology, 8600 Dübendorf, Switzerland; 3 Functional Design Laboratory, Science and Innovation Center, Mitsubishi Chemical Co., Ltd., 1000 Kamoshida-cho, Aoba-ku, Yokohama, Kanagawa 227-8502 Japan

**Keywords:** Engineering, Materials science

## Abstract

Recent advancements on electrohydrodynamic (EHD) soft pumps demonstrate their applicability to various fluid-driven systems such as soft robots, wearable devices, and stretchable electronics. In particular, fiber type EHD pumps reported more recently is a promising pumping element thanks to their versatile fibrous structure. Yet existing EHD fiber pumps are less stretchable and require sophisticated, complex fabrication equipment, implying opportunity for technology advancement. This paper presents a simplified method to create highly stretchable multifunctional fiber EHD pumps. The method employs highly compliant silicone elastomers for the fiber structure that is formed by simple dipping fabrication process. The fabricated pumps (length of 100 mm, inner diameter 4 mm, and mass 5.3 g) exhibit a high stretchability (up to 40% strain) and flow rate and pressure of 167.4 ± 7.6 mL/min (31.6 mL/min/g) and 4.1 ± 0.6 kPa (0.8 kPa/g), respectively. These performances are comparable or even higher than those of previously reported EHD pumps including fiber types. The output performance of the fabricated pumps remain constant for repeated strain cycles (0–25%, up to 2000 cycles) and bending angle up to 180° (corresponding to curvature of 0–30/m). Moreover, the pumps demonstrate unprecedented functionality as a sensor to distinguish the type of fluid inside the tube and to detect strains by reading the capacitance between the electrodes. The characterization result reveals the sensing ability of the pumps as high repeatability up to 30% strain with negligible hysteresis, which is consistent for 5000 cycles.

## Introduction

The growing demand for flexible, lightweight, and scalable pumping elements in various fields related to soft robots^1–4^, wearable systems^5–7^, and biomedical devices^8,9^ has led to the development of novel pump architectures^10–12^. Among them, one representative architecture is soft electrohydrodynamic (EHD) pumps, which have attracted significant interest due to their flexibility, compact size, light weight, and ability to generate high specific flow rates and pressures as represented in the pioneering work done by Cacucciolo et al.^13^. Recently, a further breakthrough has been made on soft EHD pumps by Smith et al.^14^ They have developed the pumps in the form of fibers in which continuous helical electrodes are embedded within the wall of thin thermoplastic polyurethane (TPU) tubing, allowing to generate fluid flow and pressure while linearly stretching the devices up to 15%. They also demonstrated the use of fiber pumps by integration with a wearable device. Here, the study also presents the opportunity for further investigation. The use of TPU as a thermoplastic requires sophisticated and complex fabrication equipment. Moreover, the relatively high modulus of TPU (Young's modulus: about 30 MPa^15^) inhibits stretchability, the characteristic that soft fiber pumps are supposed to possess.

In this study, we aim to push forward the fiber EHD pump technology. Specifically, we establish a method that simplifies the fabrication and allows the use of highly compliant materials. These materials include Dragon Skin 30 and Sylgard 184, which have Young's modulus of 0.6 and 3.9 MPa, respectively^16,17^. We show that pumps can be fabricated with silicone elastomers, a mainstream compliant material in soft robotics and stretchable electronics, and that the dipping method is applicable to realize fibrous structure with simplified fabrication equipment and associated steps. We then show that the pumps fabricated with these materials and methods can exhibit a high stretchability allowing up to 40% strain and generate flow rate of 167.4 ± 7.6 mL/min and pressure of 4.1 ± 0.6 kPa, corresponding to specific flow rate and pressure as 31.6 mL/min/g and 0.8 kPa/g, respectively. These values are almost constant even at 25% applied strain. Further, we clarify the output performance of the fiber pump under bending deformation up to 180° (corresponding to curvature of 0–30/m). The result shows that even at the bending angle of 180°, the pumping performance is not affected. Finally, we demonstrate that the fiber pumps are able to function as a sensor detecting strain of up to 30% based on capacitive sensing method. The sensing ability and high output performance thanks to the use of silicone elastomer and dipping method reveal the high potential of general concept of stretchable fiber pumps and pave the way for future developments of advanced flexible and stretchable fluidic devices with integrated sensing capabilities for applications in soft robotics, wearable technology, and stretchable electronics.

## Results and discussion

**Figure 1:**
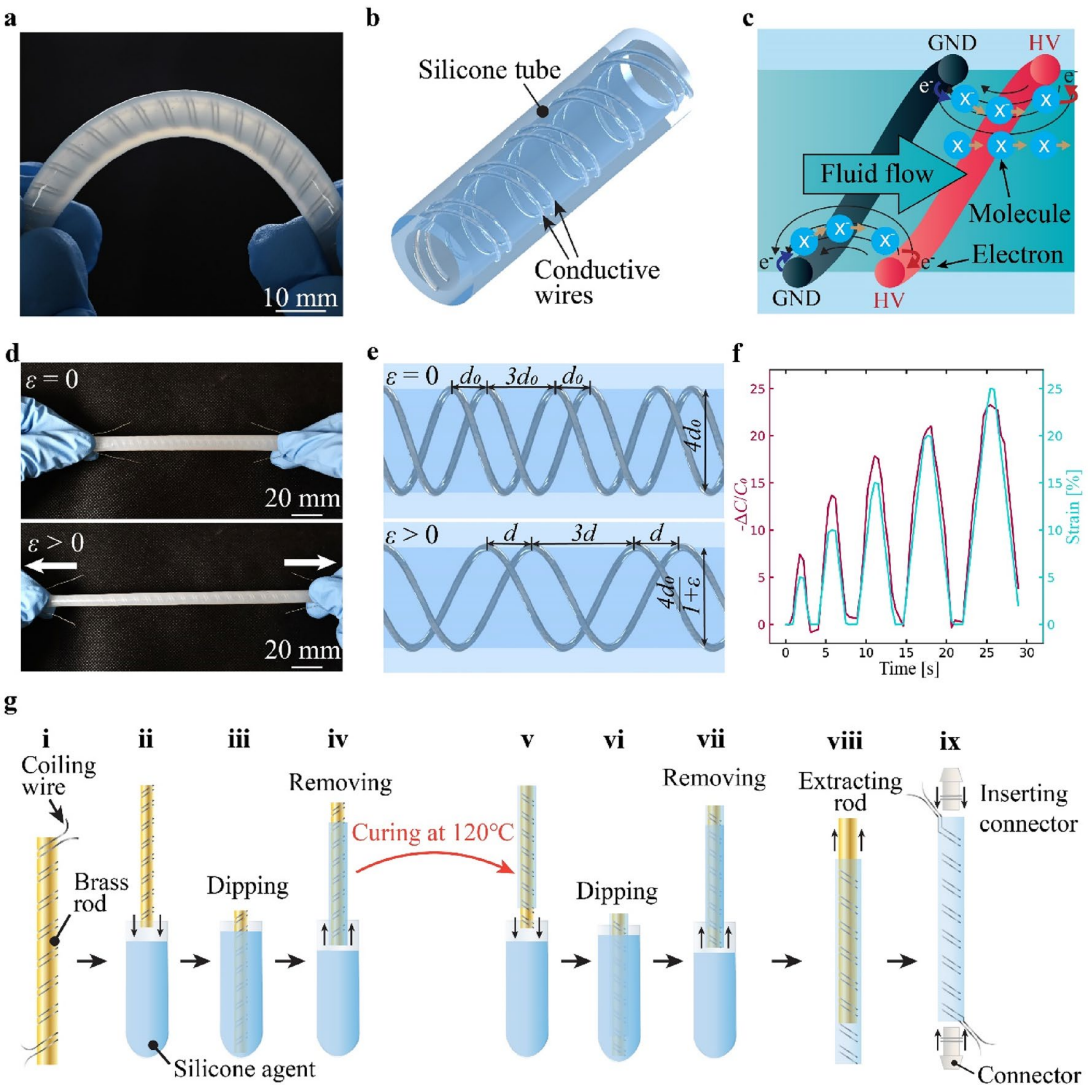
Structure, characteristics, and fabrication procedure of the silicone-based stretchable fiber pumps. (**a**) Use of silicone elastomer enables high flexibility and stretchability in the pumps. (**b**) Pumps consist of a pair of conductive wires as electrodes, embedded in a silicone tube. The electrodes are coiled in a parallel helical form. (**c**) Working principle of the pumps. Dielectric fluid molecules are ionized by electric fields generated between the electrodes produced by application of high voltage. The ionized molecules are accelerated by the electric fields, thereby creating one directional flow. (**d**) The fiber pumps are highly stretchable, allowing 40% strain. (**e**) The distance between conductive wires, designated as ${d}_{0}$ (1 mm), is increased when the pump is stretched (${d>d}_{0}$). At the time, the capacitance between the electrodes, represented as ${C}_{0}$ decreases (${C<C}_{0}$). (**f**) The capacitance change can be exploited to use the pumps as a capacitive strain sensor. (**g**) Fabrication process of the pumps. (i) Conductive wires are coiled around a brass rod. (ii, iii) The rod is then dipped into a silicone mixture. (iv) The rod covered with the silicone mixture is cured in an oven. (v, vi) The rod is inverted and dipped again. (vii) The rod with the silicone mixture is again cured in the oven. Steps (ii) through (vii) are repeated four times in total. (viii) After curing the silicone, the brass rod is then extracted, leaving a silicone tube. (ix) Connectors are inserted into both ends of the silicone tube.

The stretchable fiber pump developed in this study (Fig. 1a) is highly flexible owing to its main material, silicone elastomer. As depicted in Fig. 1b, our pumps consist of a pair of heroical conductive wires embedded in a silicone tube. This structural configuration follows the previous work done by Smith et al.^14^. Therefore, the working principle as well. The working principle of the pumps is illustrated in Fig. 1c. In the tubular structure, molecules of a dielectric fluid is ionized and accelerated by high electric field generated from helical electrode, leading to formation of one directional flow, similar to other EHD pumps^13,14,18,19^.

Thanks to the use of silicone elastomer, our pumps can be stretched over 40% strain (Fig. 1d). They possess mechanical properties akin to those of a silicone elastomer, with a slight increase in stiffness due to electrodes, which essentially function as coil springs. Therefore, the expected stress–strain behavior of the pump is expected to be non-linear, similar to that of silicone elastomers. As shown in Fig. 1e, distance between the electrodes expands under stretched state. This causes change in the capacitance between the electrodes; the capacitance decreases with increased strain. This phenomenon allows us to use the pumps as a capacitive strain sensor, as plotted in Fig. 1f (see also Supplementary Video S1). The sensor response exactly follows the rapid strain changes (50 mm/s, corresponding to a strain rate of 50%/s).

The fabrication process of the silicone-based fiber pumps is summarized in Fig. 1g. It mainly consists of electrode coiling and tube formulation by dipping of silicone. First, conductive wires used for the electrodes are coiled to a brass rod (see Supplementary Video S2). The rod is then dipped into a silicone mixture in the liquid state (see Experimental Section and Supporting Information for more detail) and cured in an oven. Different mixing ratio of the silicone mixture allows to control resulting compliance of the structure (Fig. S1). After curing the silicone, the rod is inverted and repeated the dipping process again. After four times dipping, the silicone part reaches a thickness sufficient for completely wrapping the electrodes. The rod is then removed from the silicone part to be hollow tube. Finally, connectors are jointed and pumps are obtained with length of 100 mm, inner diameter of 4 mm, and outer diameter of 6 mm (mass 5.3 g without the connectors). The core of this fabrication process is dipping that is also applied in other fibrous form such as catheters^20,21^. Given the simplicity of the fabrication process it has scalability and allow the use of not only silicone elastomers but also other polymeric materials. The diameter of the pump can be adjusted using rigid rods of different diameters. Since commercially available rods with a diameter of less than 1 mm are accessible, we presume that a submillimeter fiber pump can also be fabricated. Furthermore, the wall thickness of the fiber can be adjusted by changing the number of dips, as demonstrated in other studies involving catheters^20,21^.

**Figure 2:**
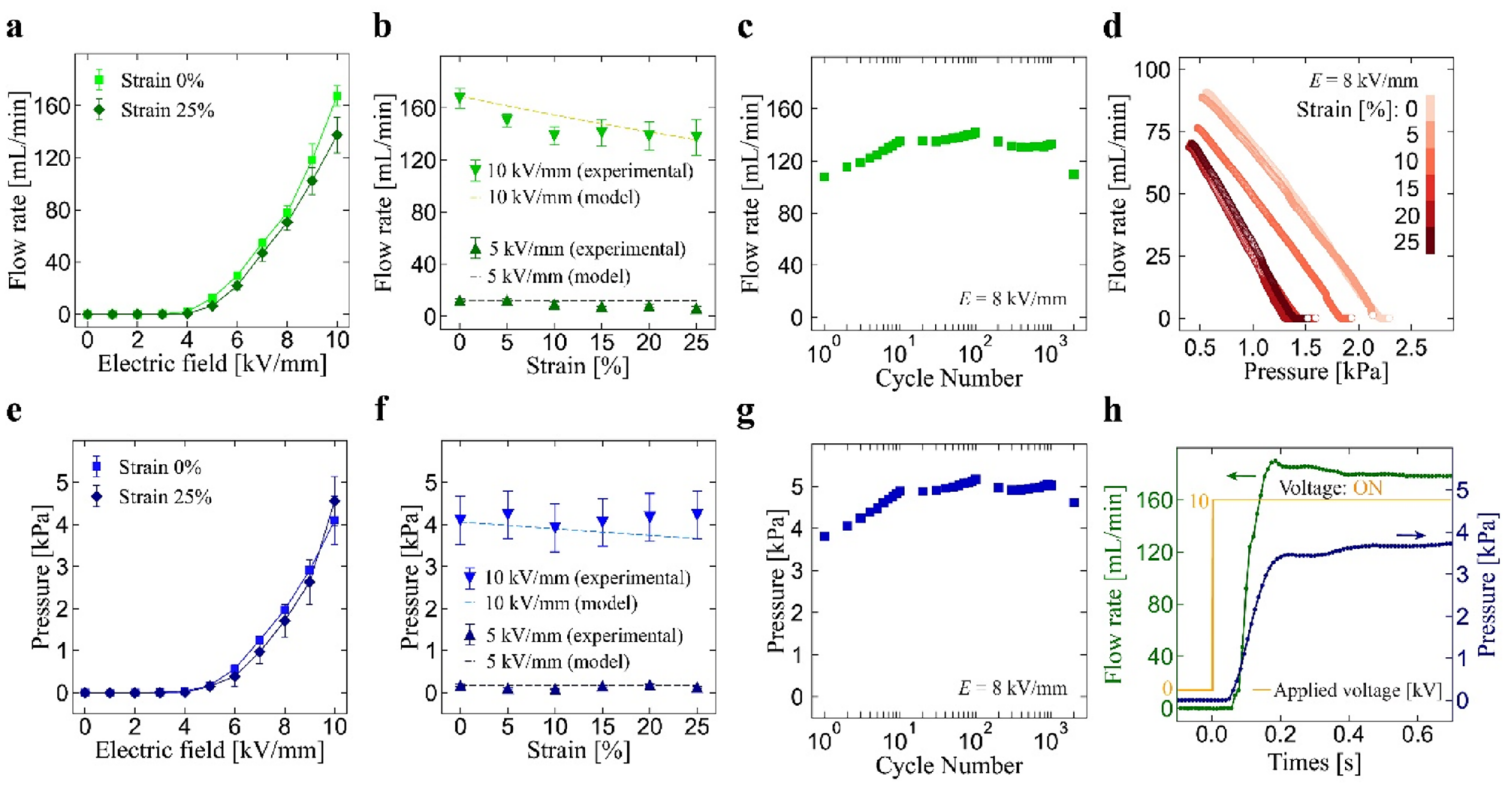
Characterization results of flow rate and pressure under different strains. Values represent the mean ± standard deviation from n = 3 samples (indicated with error bars). Flow rate under: (**a**) 0% and 25% strain at applied electric field of 0–10 kV/mm, (**b**) 0–25% strain at applied electric field of 5 and 10 kV/mm, and (**c**) cyclic strain at applied electric field of 8 kV/mm (number of cycles 2000). (**d**) Relation between the flow rate and pressure at applied electric field of 8 kV/mm. Pressure under: (**e**) 0% and 25% strain at applied electric field of 0–10 kV/mm, (**f**) 0–25% strain at applied electric field of 5 and 10 kV/mm, and (**g**) cyclic strain at applied electric field of 8 kV/mm (number of cycles 2000). (**h**) Response of flow rate and pressure at 0% strain (applied electric field 10 kV/mm).

We first asses the output performance of the pumps under different applied strains in terms of flow rate and pressure as functions of the applied voltage, as summarized in Fig. 2. In this figure, the electric field is defined as dividing the applied voltage by the gap between the electrodes in non-stretched state (1 mm). The experimental setup is shown in Fig. S2. As shown in Fig. 2a, the flow rate increased with the increasing of electric field and reaches the value of 167.4 ± 7.6 mL/min at 10 kV/mm in non-stretched state. This indicates that the output of our pumps is controllable by adjusting the intensity of electric field. The pumps are able to maintain the output even it is stretched to 25% of strain, only slight reduction is overserved (137.5 ± 13.7 mL/min at 10 kV/mm). The reason for the reduction is that enlarging gaps between the electrodes by tube elongation results in a slightly lower intensity of electric field. In order to have better understanding on the influence of the gap on the flow rate under the deformations, we built an analytical model to predict the amount of reduction in flow rate and pressure under the applied voltage and strain (see Supporting Information for more detail) and compare it with experimental data. As represented in Fig. 2b, the measured flow rate exhibits a trend that it gradually decreases with increased strain, while the model prediction fits well. This indicates that the designing and performance tuning is possible for silicone-based fiber pumps.

The measured flow rate of the pumps for cyclic applied strain (0–25%) is plotted in Fig. 2c. Overall, the pumps are able to maintain the output for 2000 strain cycles. From a local point of view, the flow rate is stabilized after 10 cycles. At this point, it is believed that the ionized fluid filled the entire system, causing the increase in flow rate to cease. Figure 2d shows the relation between the flow rate and pressure. The data exhibits a linear relationship between the two, and as expected, the output decreased when strain is applied. Figure 2e–g plots characterization results of the pumps in terms of output pressure. Similar to the case of flow rate, the pressure increased with the increasing electric field and reaches the value of 4.1 ± 0.6 kPa, at 10 kV/mm in non-stretched state. In the stretched state, again only slight reduction is observed (4.1 ± 0.6 kPa). The pressure under different stains (Fig. 2f) and cyclic strains (Fig. 2g) exhibit similar trend to the case of flow rate. However, slight increase in the flow rate at larger strains are observed. This may have resulted from measurement error. Figure 2h shows the response of flow rate and pressure at 10 kV/mm. The response started within 0.1 s when voltage is applied. In evaluating the performance of the pump, the diameter of the silicone tube emerges as a crucial factor influencing both the flow rate and pressure, as reported by Smith et al.^14^. Their findings indicate that reducing the pump diameter enhances the maximum pressure while decreasing the maximum flow rate. It is anticipated that our pumps will exhibit similar characteristics, given their fundamentally identical structural design.

**Figure 3:**
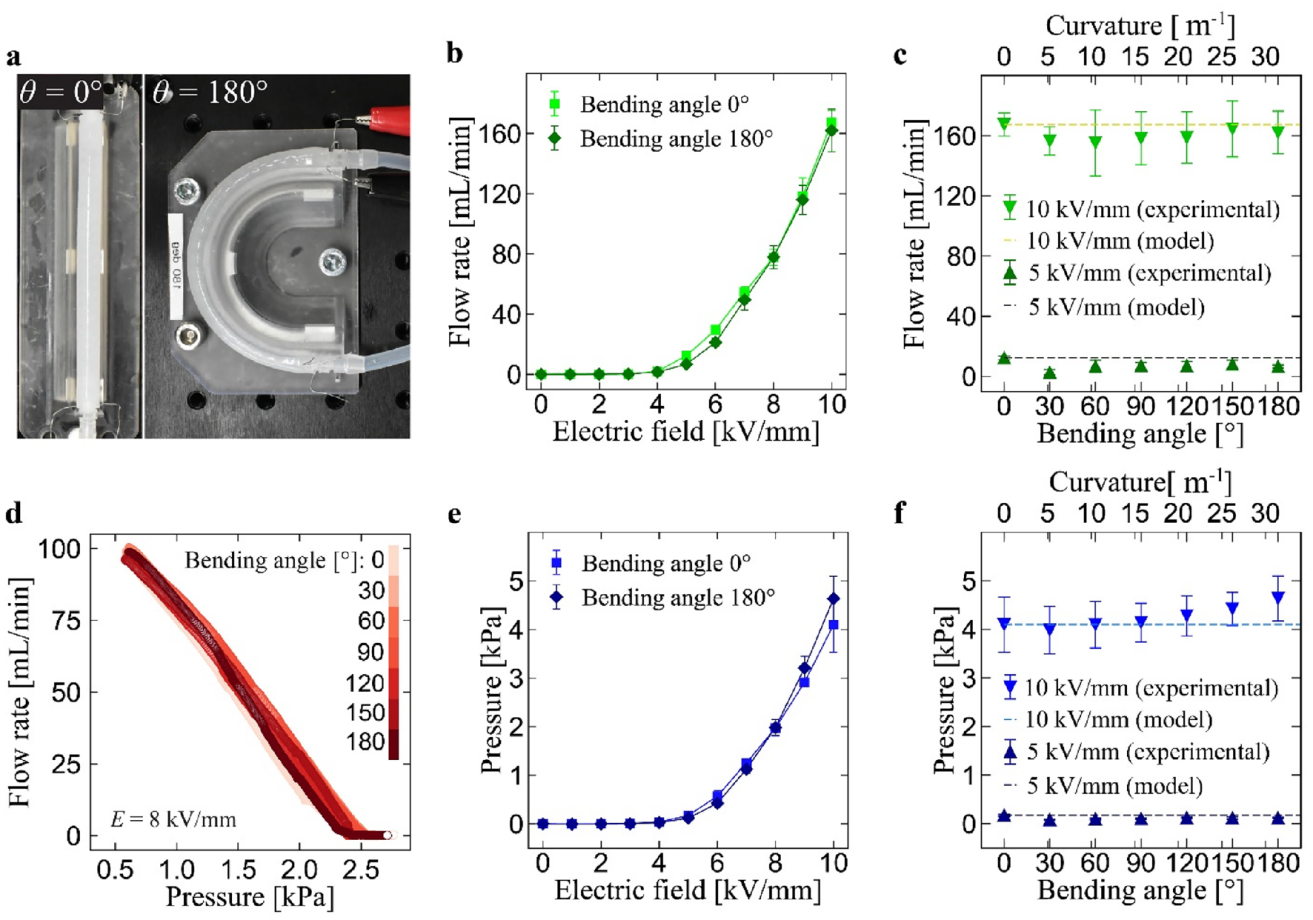
Characterization results of flow rate and pressure under different bending angles. Values represent the mean ± standard deviation from n = 3 samples (indicated with error bars). (**a**) Pumps placed in holding setup to apply bending angle of 0 and 180°. (**b**) Flow rate as a function of the applied electric field for bending angle of 0 and 180°. (**c**) Flow rate as a function of the bending angle at the electric field of 5 and 10 kV/mm. (**d**) Relationship between flow rate and pressure under the electric field of 8 kV/mm for the bending angle from 0 to 180° (**e**) Pressure as a function of the applied electric field for bending angle of 0 and 180°. (**f**) Pressure as a function of the bending angle at the electric field of 5 and 10 kV/mm.

The high compliance of the pump structure allows it to bend. We then characterize the output performance of the pumps under different bending angles in terms of flow rate and pressure as functions of the applied voltage, as shown in Fig. 3. In this test, the bending angle applied to the pump is changed with 30° of step increment. Each bending angle is applied using a holder with different radius (curvature). The value of radius and curvature of the holder for the bending angles used in this experiment is summarized in Table S1. Figure 3a shows the holder used to achieve 0° and 180° of bending angle. The result has revealed that the flow rate experiences only a slight reduction even the bending angle is 180° (Fig. 3b). The same applies to the other different bending angles (Fig. 3c). The reason is that the net intensity of electric fields in the silicone tube does not change under bending state, where the gap between the electrodes widens on the outside of the neutral plane of bending deformation and narrows on the inside, canceling each other out as a whole (see Fig. S3 for a schematic representation). The data presented in Fig. 3d supports the hypothesis, where traces of the relationship between the flow rate and pressure always on the similar pathway regardless of the amount of bending angle. This is further confirmed with the measured pressure shown in Fig. 3d–f, in which the pressure value remains almost constant for different bending angles.

**Figure 4:**
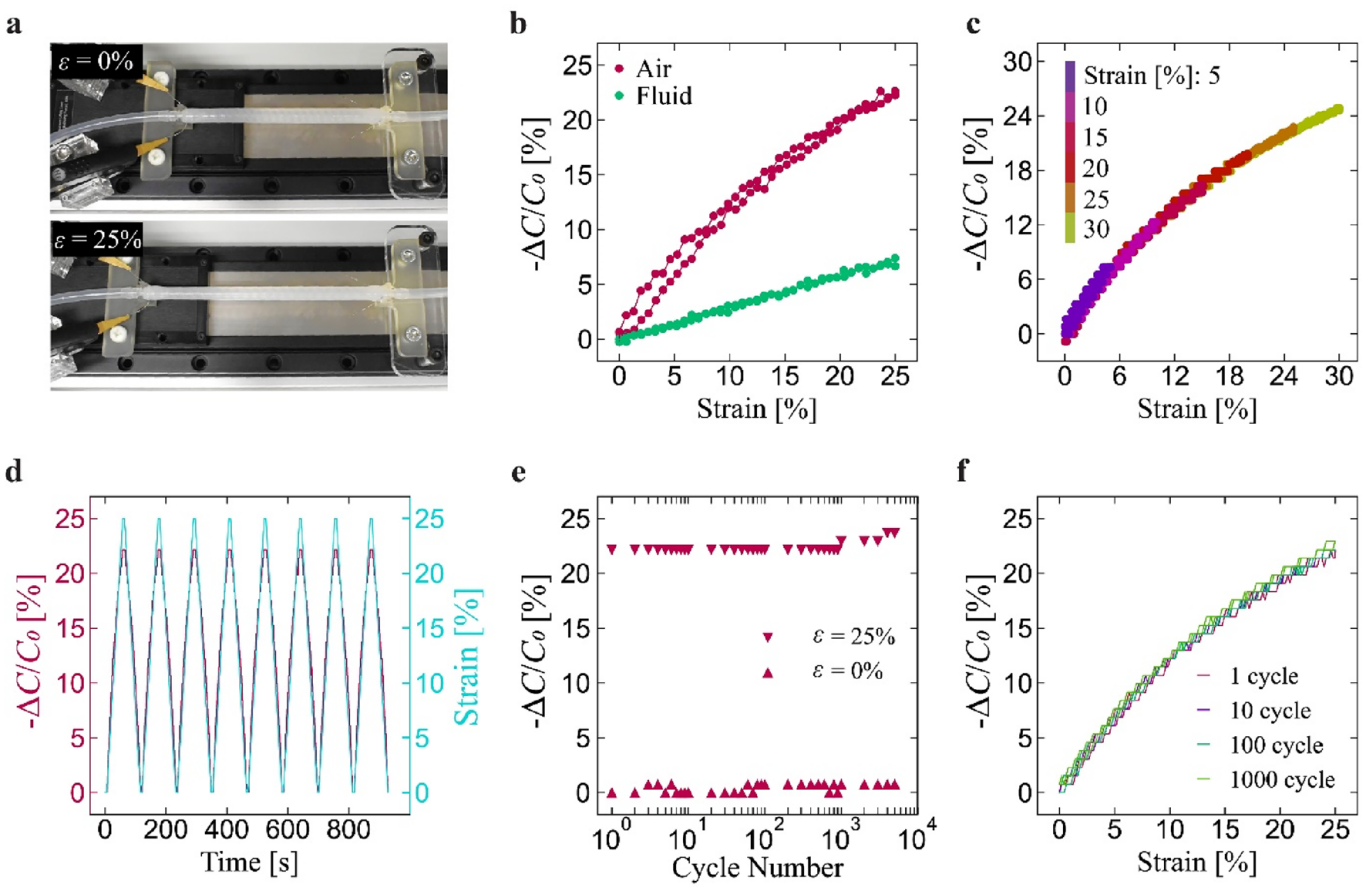
Characterization results of sensing ability of the pumps. (**a**) Experimental setup to apply linear strain to the pumps. Capacitance changes: (**b**) during a single-cycle strain (up to 25%) when the tube is filled with air or fluid, (**c**) during a single-cycle strain with 5% strain increments up to 30%, (**d**) with strain profile over the first eight cycles, (**e**) at 0% and 25% strain as a function of number of cycles, and (**f**) at 1, 10, 100, and 1000th cycles.

As mentioned previously, the silicone-based fiber pumps can function as a sensor by reading the capacitance change between the electrodes while it is stretched. It is worth to note that, due to the cancellation effect discussed earlier, the capacitance remains constant during the bending states. We next characterize the sensing ability of the pumps using an experimental setup shown in Fig. 4a, where up to 30% of linear strain is applied to the pump using a motorized stage. Figure 4b represents a typical behavior of the sensor response. The capacitance decreases with increasing the strain. The sensing behavior is expected to remain constant, regardless of the diameter of the electrodes and their initial distance, given that strain is an essential parameter. However, the absolute value of capacitance varies with diameter and distance. Extremely thin wires, representing a relatively small electrode area, or extremely long distances result in significantly small capacitance values. This requires high precision on the sensor value-acquiring side, and in some cases, the sensor response may be undetectable. Therefore, it is crucial to set the diameter and distance between the electrodes such that the absolute capacitance value is appropriate for the measurement accuracy of the system.

Interestingly, the amount of capacitance change is different among the material filled in the tube: air and dielectric fluid. This is a result contrary to intuition as the capacitance is normalized to its initial value, so the response is expected to be the same regardless of the material inside the tube. One potential reason for this is current leakage from the electrodes and the change in resistance between them. This is particularly pronounced due to the significantly higher conductivity of this fluid compared to air. Specifically, the conductivity of the liquid media is 61.0 × 10^−9^ S/m, whereas the air conductivity is − 1.2 × 10^−12^ S/m^22,23^. Leakage current leads to smaller capacitance. As the pump elongates and the distance between the electrodes increases, the leakage current decreases. This allows the electrodes to retain more charges, consequently increasing the capacitance. However, the expanding distance between the electrodes also contributes to the reduction in capacitance. The interplay of these two phenomena results in a sensor response with the fluid medium that differs from that with the air. Nevertheless, this phenomenon also hints at the possibility of using the pump not only for detecting strains but also for identifying the type of materials.

Regarding the sensing capability of the pump, both strain and fluid type can be independently detected by measuring the inductance of the electrode, which can be considered equivalent to that of a solenoid. The inductance of a solenoid is expressed by the following equation:

1 $$\begin{array}{*{20}c} {L = \frac{{\mu N^{2} }}{l}\pi r^{2} } \\ \end{array}$$

Here, $\mu$ represents the magnetic permeability of the core (in our case, fluid media inside the tube), $N$ is the number of turns, $l$ is the length of the solenoid, and $r$ is its diameter. Given that $N$ is constant and the change in $r$ is negligible compared with $l$, the inductance decreases as $l$ increases. Based on this principle, it is possible to detect the strain of the fiber pump containing fluid. As most fluids have low magnetic susceptibility, their magnetic permeability μ remains nearly constant (e.g., 1.256637 × 10^−6^ and 1.256627 × 10^−6^ for air and water, respectively^24^). This implies that the inductance of the pump is insensitive to the fluid, allowing the detection of strain independent of the type of fluid.

All the subsequent experimental results (Fig. 4c–f) are obtained under air-filled condition. The pumps are also able to detect strains for different amount of cyclic up to 30% (Fig. 4c), with almost no hysteresis observed. The sensor response also accurately matches the applied strain and exhibits high repeatability (Fig. 4d), which is consistent for up to 5000 cycles (Fig. 4e). In the cyclic test, the drift error (see Experimental Section for more detail) at final strain cycle is 0.8% at 0% strain and 6.5% at 25% strain. These results indicate the high durability and repeatability of the pump as a sensor.

## Concluding remarks

In this work, we have developed silicone-based multifunctional stretchable fiber pumps. Our stretchable fiber pumps, constructed from silicone elastomer, exhibit a high stretchability enabling stretch of up to 40% strain without sustaining damage. This level of resilience has not been demonstrated in fiber pumps in the literature. The simplicity of the fabrication process that we have established, based on a dipping process, is scalable and may allow using other polymeric materials. Notably, the performance of our pumps observed as 167.4 ± 7.6 mL/min (31.6 mL/min/g) and 4.1 ± 0.6 kPa (0.8 kPa/g), is comparable to outperforms or compare that of previously reported EHD pumps including fiber types (e.g., 6.0 mL/min/g and 14.0 kPa/g in ref.^13^, 140 mL/min/g and 46 kPa/g in ref.^14^).

The results obtained through this study, suggest that our fiber pumps could serve as an efficient replacement for other types of pumping systems. Moreover, the performance of our pumps remains almost constant regardless of the magnitude of applied strain (up to 25%) and bending angle (up to 25%) and cyclic strain (number of strain cycles 2000). Additionally, given that the generated flow rate and pressure can be accurately estimated even when the pumps are stretched or bent, they can be specifically designed to meet various operational needs. Our findings also indicated that the sensor could operate effectively in both air-filled and fluid-filled environments, even under high-strain conditions. Our pumps maintain their sensing functionality while stretching up to 30% strain and experiencing strain cycles of up to 5000, demonstrating high repeatability, durability, and low hysteresis. Consequently, our fiber pumps present high performance, compact size, and robust sensing functionality. Such multifunctional characteristics are expected to advance the developments in research areas such as soft robotics, wearable technology, and stretchable electronics, where these attributes are particularly demanded. Specifically, the integration of our fiber pumps with hydraulic soft actuators could pave the way for the creation of a robotic limb, skin, or active textile. Such elements can provide dynamic movements for a range of applications, including mobile robots, manipulators, and human assistive suits, while simultaneously sensing their deformations for feedback control and monitoring.

## Methods

### Materials

Conductive wire, tinned copper wire with a diameter of 0.32 mm, was purchased from Kyowa Harmonet. The diameter of 0.32 mm is optimal for this wire, as a thicker gauge would be too stiff for stretching, while a thinner one would not adhere properly to the tube. Silicone elastomers, Sylgard 184 and Dragon Skin 30, were purchased from Dow Corning, and Smooth-On, respectively. The dielectric fluid (polyvinyl chlorid : dibutyl adipate = 0.2 wt%: 99.8 wt%) was supplied from Mitsubishi Chemical. This dielectric fluid was chosen based on our previous study^22^.

### Fiber pump fabrication

Electrodes were formed by coiling the wires along with a brass rod with the 4 mm diameter and 100 mm length (purchased from Hikari Mall). A 3D printed mold made of a UV-curable resin (Clear Resin v4, Formlabs) was used as a zig to support the coiling process (the mold was printed using Form3, Formlabs). The gap between the wires was set to 1 mm with 3 mm spacing. These values followed the reference in the literature^25^. The rod with coiled wires was dipped to a silicone mixture made of Sylgard 184 and Dragon Skin 30 mixed in weight ratio of 1:3. The rod was then cured in an oven at 120 °C for 10 min. The dipping process was repeated at four times to form tube. At the final step, curing condition was set to 120 °C for 20 min. The rod was then removed and cured silicone tube with helical electrodes was obtained. Microfluidic connectors were attached at the both ends of the tube.

### Flow rate and pressure measurements

Fiber pump was activated by high voltage amplifier (HOPP-10P(A), Matsusada) powered by a DC power supply (PMX32-2QU, Kikusui), allowing to apply a desired voltage up to 10 kV. In the experimental setup used for this measurement (Fig. S2), the liquid flow was circulated by a closed loop flow circuit. Flow rate was then measured by ultrasound flow rate sensor (FD-XS8, Keyence) and a multimeter (2100/100, Keithley) running at 100 Hz. The pressure generated from the pump was measured by a pressure sensor (ADP5120, Panasonic) and the multimeter running at 100 Hz. During the measurement, the flow circuit is blocked by pinch valve (MPPV-4, Resolution air) controlled by PLC (Arduino mega, Arduino). In every test, the pump was activated for 30 s and average of 5 s was taken as measured data. During the experiment, all the signals were communicated and recorded with PC.

### Capacitance measurement

The capacitance of the pump was measured by an LCR meter (LCR6002, Gwinstek) with sampling frequency 1000 Hz. The strain was applied to the pump by a motorized linear stage (X-LRT1000DL-E08C, Zaber). During the experiment, the capacitance and the strain (i.e., stroke position of the stage) were recoded to PC.

### Cyclic strain test

The pump was fixed to the motorized linear stage and repetitively stretched for 5000 times at the same speed of 187.5 mm/s from its initial position to 25% strain (25 mm distance). Drift error (${D}_{e}$) at 5000th cycle was obtained as:

2 $$\begin{array}{*{20}c} {D_{e} \left[ \% \right] = \left( {\frac{{C_{5000} }}{{C_{1} }} - 1} \right) \times 100} \\ \end{array}$$

where *C*_5000_ and *C*_1_ was the capacitance at 5000th cycle and first cycle, respectively.

### Supplementary Information

Supplementary Information.

Supplementary Video S1.

Supplementary Video S2.

## Data Availability

All data generated or analyzed during this study are included in this published article and its supplementary information file.
