# Supplementary material for: Silicone-based highly stretchable multifunctional fiber pumps
Source: Sci Rep. 2024 Feb 26;14:4618. doi: 10.1038/s41598-024-55472-0 (PMC10897224; doi:10.1038/s41598-024-55472-0)
Supplement: Supplementary file 1 — Supplementary Information. [file 41598_2024_55472_MOESM1_ESM.docx]

Supplementary Information

Material composition ratio for suitable stiffness

We would like to get a soft material, but return to original length. For selecting the suitable material and composition ratio, we selected two silicone elastomer Dragon Skin 30 (Dragon), and Sylgard 184 (Syl). Dragon is softer than Syl. We know Sylgard 184 100% is too stiff and Dragon Skin^TM^ 30 100% is too soft not to return to original position. Therefore, we changed to material composition ratio for suitable stiffness and return to original length. Finally, Syl : Dragon = 2.5:7.5 is suitable.

**
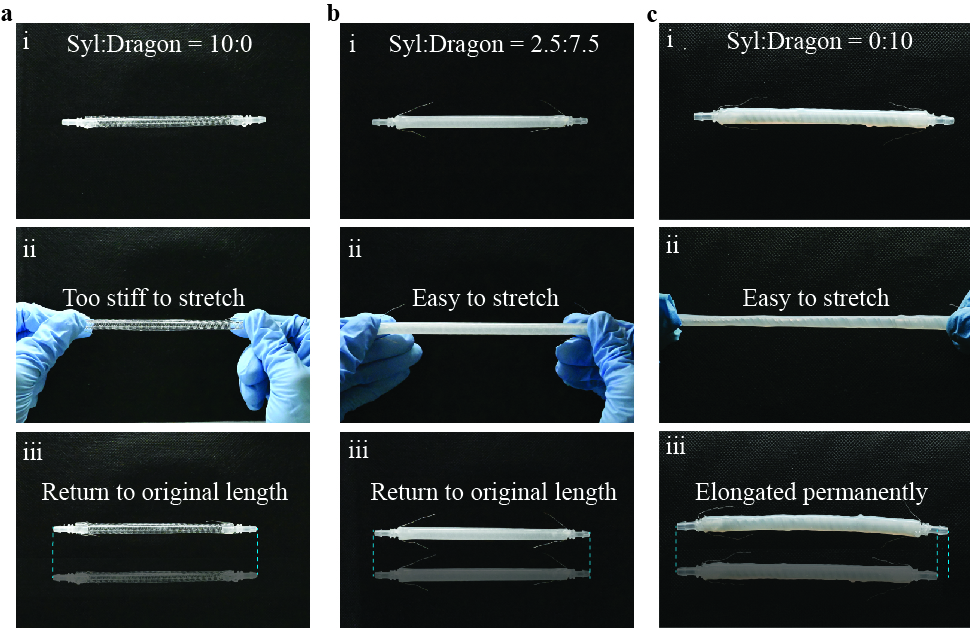
**

**Figure S1.** Elongation test at max strain. Material composition of Syl : Dragon is a) 10:0, b) 2.5:7.5, c) 0:10.

**
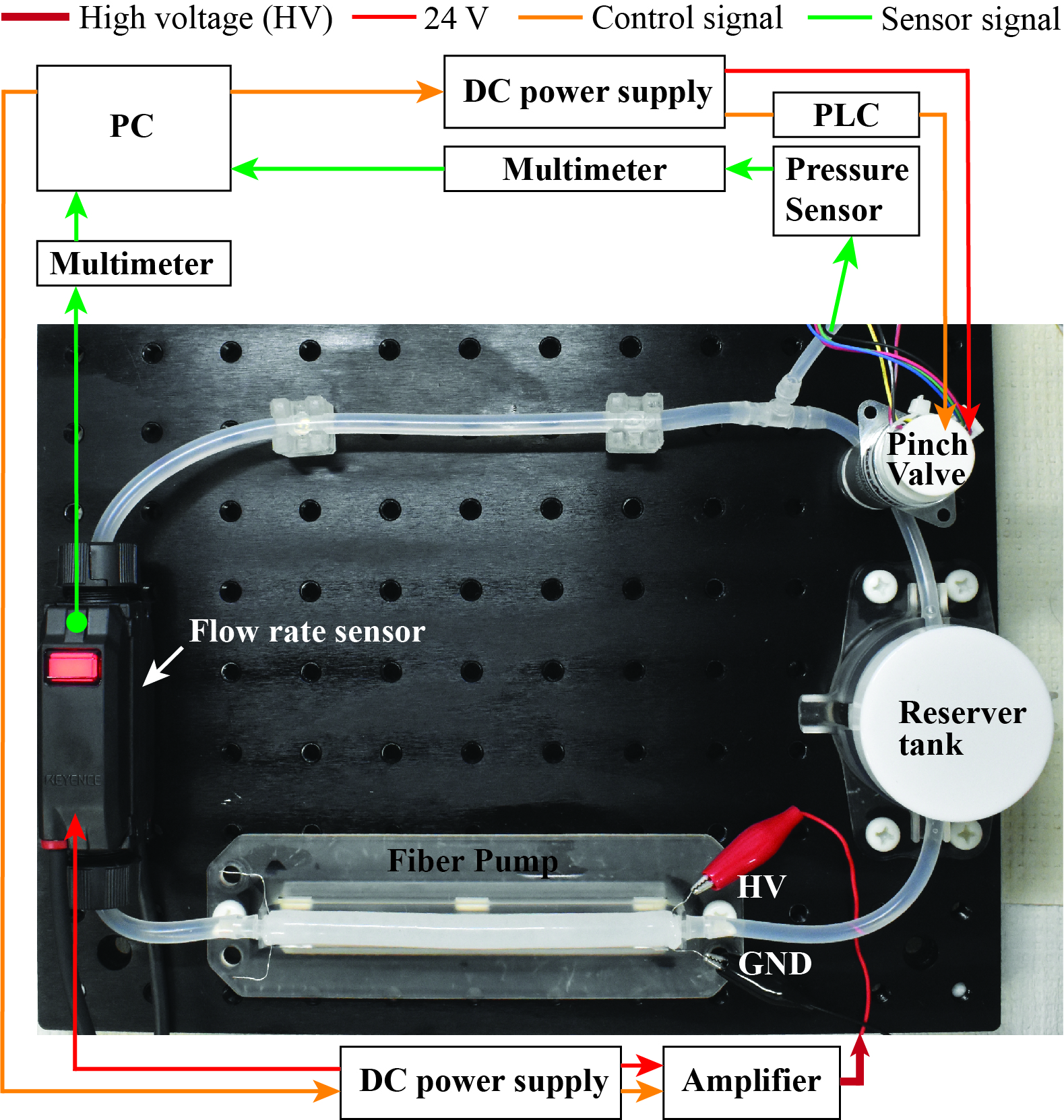
**

**Figure S2.** Experimental setup for pressure and flow rate.

**Table S1.** Radius, curvature values corresponding to different bending angles.

| Bending angle [°] | Radius [mm] | Curvature |
| --- | --- | --- |
| 30 | 190 | 0.005 |
| 60 | 96 | 0.010 |
| 90 | 63.7 | 0.016 |
| 120 | 47.75 | 0.021 |
| 150 | 38.35 | 0.026 |
| 180 | 15.9 | 0.031 |

Pump’s performance model

At the non-stretched state, ideal pressure (without any impedance), $P_{0}$ is written in Equation 1 (according to ^1^).

$$\begin{aligned} P_{0}=k{E_{0}}^{2}=k\left( \frac{V}{d_{0}} \right)^{2}\#\left( S1 \right) \end{aligned}$$

Where, $k$ is constant. $E_{0}$ is electric field at non-stretched state. Therefore, pressure at stretched state, $P_{\varepsilon}$ can be written below.

$$\begin{aligned} P_{\varepsilon}=k{E_{\varepsilon}}^{2}=k\left( \frac{V}{d} \right)^{2}=k\left\{ \frac{V}{d_{0}\left( 1+\varepsilon\right)} \right\}^{2}=\frac{P_{0}}{\left( 1+\varepsilon\right)^{2}}\#\left( S2 \right) \end{aligned}$$

According to the literature ^2^, flow rate in non-stretched state ($F$) can be described as follows:

$$\begin{aligned} F=F_{0}\left( 1-\frac{P}{P_{0}L_{0}} \right)\#\left( S3 \right) \end{aligned}$$

Also, $F_{i}$ can be described from the point of Hagen- Poiseuille type flow.

$$\begin{aligned} F=\frac{P}{Z_{S}+Z_{f}L}\#\left( S4 \right) \end{aligned}$$

Where, $Z_{S}$ is fluidic impedance, $Z_{f}$ is the impedance per unit length. By solving Equation S3 and S4 as $P$, following equation is obtained.

$$\begin{aligned} F=\frac{F_{0}P_{0}L}{P_{0}L+F_{0}\left( Z_{S}+Z_{f}L \right)}\#\left( S5 \right) \end{aligned}$$

By applying $P_{\varepsilon}$ of Equation S2 to $P_{0}$ of Equation S5, flow rate under strain ($F_{\varepsilon}$) is obtained.

$$\begin{aligned} F_{\varepsilon}=\frac{F_{0}P_{\varepsilon}L}{P_{\varepsilon}L+F_{0}\left( Z_{S}+Z_{f}L \right)}\#\left( S6 \right) \end{aligned}$$

Parameters ($P_{0}$, $F_{0}$, $Z_{S}$, $Z_{f}$) used model are described in Table S2.

**Table S2** Model parameter

| *P_0_* [kPa] | *F_0_* [mL min^-1^] | *Z_s_* | *Z_f_* |
| --- | --- | --- | --- |
| 6.7 | 429 | 0.012 | 0.012 |

Influence of bending on electric field

In bent state, the gaps between electrodes is shown in Figure S4. The average gaps ($d_{ave}$) is described in following equation.

$$\begin{aligned} d_{ave}=\frac{1}{x}\int_{d_{0}\left( 1-2\sin\frac{\theta}{2} \right)}^{d_{0}\left( 1+2\sin\frac{\theta}{2} \right)} dx=d_{0}\#\left( S1 \right) \end{aligned}$$

Where, $x$ represents diameter of the pump. $d_{0}$ is the gap at the tube center.$\theta$ is two of the electrode angle. According to Equation S1, $d_{ave}$ remains constant and equals to $d_{0}$. Consequently, electric field also maintains constant value.

**
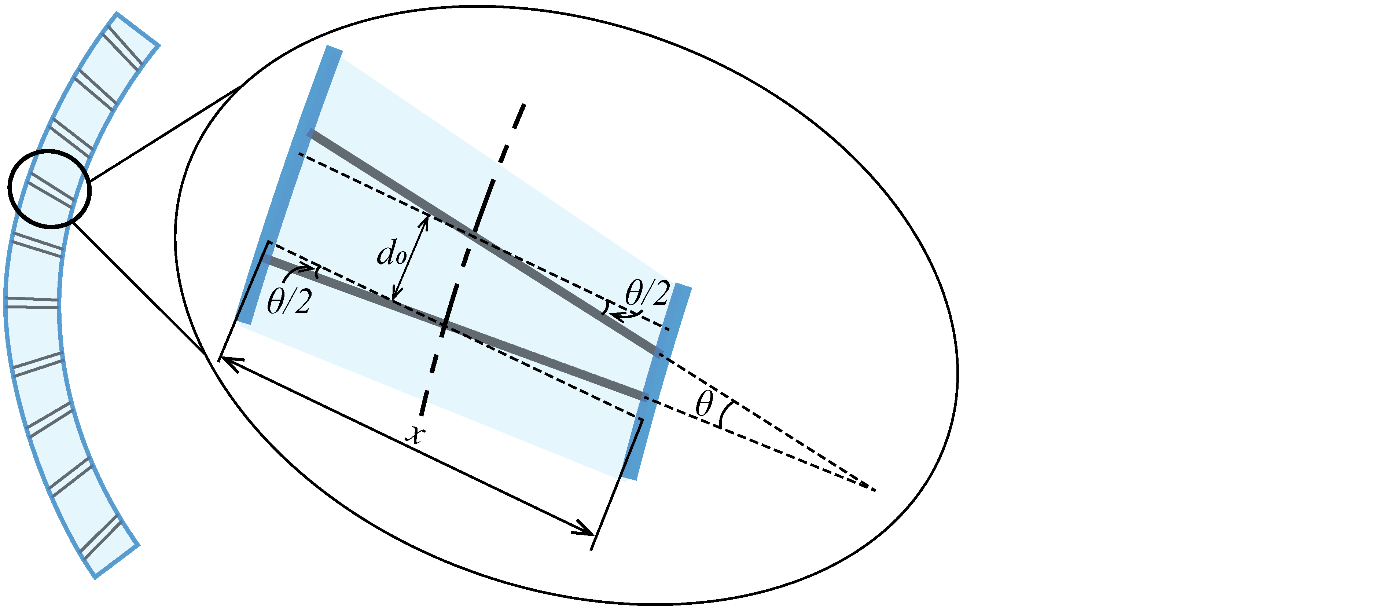
**

**Figure S3.** Cross-section view of pumps in bent state.

1. Smith, M., Cacucciolo, V. & Shea, H. Fiber pumps for wearable fluidic systems. *Science* **379**, 1327–1332 (2023).

2. *Electrokinetics and Electrohydrodynamics in Microsystems*. (Springer Vienna, 2011). doi:10.1007/978-3-7091-0900-7.
